# Can an Acceptance and Commitment Therapy-Based Smartphone App Help Individuals with Mental Health Disorders Quit Smoking?

**DOI:** 10.1155/2024/1055801

**Published:** 2024-06-21

**Authors:** Margarita Santiago-Torres, Kristin E. Mull, Brianna M. Sullivan, Judith J. Prochaska, Michael J. Zvolensky, Jonathan B. Bricker

**Affiliations:** ^1^Division of Public Health Sciences, Fred Hutchinson Cancer Research Center, Seattle, Washington, USA; ^2^Stanford Prevention Research Center, Department of Medicine, Stanford University, Palo Alto, California, USA; ^3^Department of Psychology, University of Houston, Houston, Texas, USA; ^4^HEALTH Institutive, University of Houston, Houston, Texas, USA; ^5^University of Texas MD Anderson Cancer Center, Houston, Texas, USA; ^6^Department of Psychology, University of Washington, Seattle, Washington, USA

## Abstract

**Background:**

Individuals with mental health disorders face major barriers in accessing smoking cessation care, often due to the stigmas associated with mental disorders and addiction. Consequently, accessible population-based smoking cessation interventions are needed for this vulnerable group.

**Objective:**

This secondary analysis utilized data from a 12-month randomized trial to examine whether an acceptance and commitment therapy-based app (iCanQuit) demonstrated greater efficacy, engagement, and satisfaction compared to a United States (US) Clinical Practice Guidelines-based app (QuitGuide) in helping adults with mental health disorders quit smoking.

**Materials and Methods:**

Participants self-reported having bipolar disorder or schizophrenia, or screened positive for depression, generalized anxiety, panic disorder, posttraumatic stress disorder, or social anxiety. We compared the primary outcome of self-reported 30-day cigarette abstinence at 12 months between iCanQuit (*n* = 770) and QuitGuide (*n* = 785) using complete-case and multiple imputation analyses and compared engagement and satisfaction between arms. Mediation analyses were conducted to examine whether the intervention apps functioned by reinforcing hypothesized mechanisms of action, namely, acceptance of triggers to smoke and through app engagement.

**Results:**

Participants represented all 50 US states and had 30.2% non-White or Hispanic backgrounds. Among participants with any mental health disorder, iCanQuit demonstrated higher 30-day cigarette abstinence than QuitGuide at 12 months (complete-case: 24.4% vs. 20.4%, *P*=0.04; multiple imputation: 24.6% vs. 20.4%, *P*=0.04). A comparable effect size was observed in iCanQuit participants with bipolar disorder or schizophrenia compared to QuitGuide, albeit not statistically significant (multiple imputation: 27.1% vs. 20.9%; *P*=0.06). iCanQuit's cessation efficacy was mediated by acceptance of emotions triggering smoking (*P* < 0.001) and app engagement (*P* < 0.001). iCanQuit was more satisfying than QuitGuide (88.5% vs. 77.2%; *P* < 0.001).

**Conclusions:**

In the largest known study of ACT for smoking cessation among adults with mental health disorders, the smoking cessation, engagement, and satisfaction outcomes were all significantly greater with iCanQuit than QuitGuide. Acceptance of emotions triggering smoking and iCanQuit app engagement were important mechanisms of efficacy. This trial is registered with NCT02724462.

## 1. Introduction

Cigarette smoking is a leading cause of premature death, responsible for approximately 480,000 deaths in the United States (US) annually [1, 2]. Despite smoking rates among the US general adult population reaching a record low of 11.5% in recent years [3], this positive trend has not extended to individuals with mental health disorders. In fact, in the US, individuals with mental health disorders are nearly twice as likely to report cigarette smoking when compared to those without mental health disorders (28.2% vs. 15.8% for past-month cigarette smoking) [4, 5]. Such disparities in smoking rates among people with mental health disorders underscore the limitations of existing initiatives targeting the general public. Developing and disseminating treatments that effectively decrease smoking among individuals with mental health disorders can mitigate smoking-related inequities.

Although people with mental health disorders share a similar desire to quit smoking compared to people without such disorders, their success rate is 25% lower [6, 7]. Various factors contribute to such challenges in quitting, including elevated negative affect states and emotion dysregulation deficits [8, 9]. For example, some people may smoke more during heightened levels of emotional distress [10]. Study of previously secret internal documents has revealed that the tobacco industry engaged in a variety of direct and indirect efforts to promote a self-medication hypothesis of nicotine as medicine, which likely has contributed to the slow decline in smoking prevalence in people with serious mental illness, such as schizophrenia [11]. Nicotine affects dopamine release, providing temporary pleasure and reinforcing the perception that smoking alleviates mental health symptoms, despite actually worsening these symptoms in the long-term [12, 13, 14, 15]. Considering the profound influence of negative affect states and regulatory challenges in managing symptoms of mental illness [9, 16, 17, 18], smoking cessation treatments that address these inner psychological experiences could hold promise.

One approach to addressing negative affect states is acceptance and commitment therapy (ACT) [19]. Unlike standard therapy [20], ACT focuses on helping individuals accept cravings that trigger smoking rather than relying on avoidance strategies to eliminate uncomfortable internal physical sensations, thoughts, or emotions that trigger smoking. This method has shown promise in smoking cessation interventions targeting the broader adult population [21, 22, 23]. Research focused on applying ACT or acceptance principles via in-person delivery modalities to smoking cessation for persons with anxiety and depression has shown initial promise [24, 25], although less positive results have been evident for those with serious mental health disorders [26].

Individuals with mental health disorders also face complex barriers to accessing smoking cessation care due to a myriad of factors including limited resources and the stigmatizations of mental disorders and addiction [27, 28]. There is also widespread evidence that smoking is often not addressed (e.g., assessed or integrated into treatment) in mental health treatment facilities [29, 30]. Moreover, mental health professionals may lack knowledge of and experience with tobacco treatments for encouraging and supporting cessation for this population [30, 31]. There is a need for accessible and efficacious population-based interventions that can easily be integrated within existing care models [5, 7].

A strategy for reducing accessibility barriers is to freely offer remotely delivered cessation treatments [32]. Although evidence-based telephone-delivered smoking cessation interventions can be efficacious [33], they have yielded relatively low cessation rates among people with mental health disorders, typically ranging from 3% to 18% [33, 34, 35, 36, 37]. Moreover, telephone-delivered smoking cessation interventions have demonstrated limited reach. For instance, state quitlines only reach only 1%–2% of all US adults who smoke [38, 39]. Digital interventions, such as smartphone applications (“apps”), offer an alternative approach, with the potential for broader reach and greater acceptance [40, 41]. Moreover, smartphone apps can be discreetly accessed, potentially reducing stigma often experienced by individuals with mental health disorders [42].

Presently, there is limited evidence for the efficacy of smartphone apps delivering behavioral smoking cessation interventions for individuals with mental health disorders. Most studies have been limited to small pilot randomized controlled trials (RCTs) and single-arm studies [43, 44, 45, 46, 47]. For instance, a pilot RCT compared the preliminary efficacy of the “Learn to Quit” smoking cessation app, which is based on ACT and was specifically designed for individuals with serious mental illness (i.e., bipolar disorder, schizophrenia, and major depression disorder), to the National Cancer Institute-developed “QuitGuide” app that follows the US Clinical Practice Guidelines (USCPG) for smoking cessation [47]. In this study of 62 adults with serious mental illness, at 4 months, the 30-day abstinence rates were 12% for Learn to Quit versus 3% for QuitGuide (*P*=0.23). The quit rates in the Learn to Quit arm were modest and not statistically significantly different from the QuitGuide likely due to lack of power.

Our research group conducted a full-scale Phase III two-arm RCT to assess the efficacy of an ACT-based smartphone app, named iCanQuit, for smoking cessation in the general population. In this 12-month trial, we compared iCanQuit to QuitGuide in a large and diverse sample of 2,415 adults from all 50 states in the US representing various geographic and racial/ethnic backgrounds [48]. At 12 months, iCanQuit was 1.5 times more efficacious than the QuitGuide for smoking cessation (OR = 1.49; 95% CI: 1.22, 1.83)—with quit rates of 28.2% and 21.1%, respectively. We also observed that the effect of the intervention on quitting cigarette smoking was mediated by changes in ACT core processes, specifically, acceptance of internal physical sensations and emotions that cue smoking [49].

In this secondary analysis, we used data from the iCanQuit parent RCT to evaluate the efficacy, engagement, and satisfaction of iCanQuit relative to QuitGuide in individuals with mental health disorders—defined as those who either self-reported bipolar disorder or schizophrenia or screened positive for depression, generalized anxiety, panic, posttraumatic stress disorder (PTSD), or social anxiety. We hypothesized that iCanQuit—which emphasizes acceptance of internal cues triggering smoking—would demonstrate greater efficacy, engagement, and satisfaction in helping adults with mental health disorders to quit cigarette smoking as compared to the QuitGuide, which promotes the avoidance of cues to smoke.

## 2. Materials and Methods

### 2.1. Procedures

We used data from a 12-month randomized trial that compared the ACT-based app iCanQuit to the USCPG-based app QuitGuide for cigarette smoking cessation among 2,415 adults [48, 49, 50, 51, 52, 53, 54]. Interested individuals were eligible to participate in the parent trial if they reported smoking five or more cigarettes per day, had daily access to a smartphone, and expressed a desire to quit smoking in the next 30 days. Those who reported not being able to read English, presently receiving treatment for smoking cessation, using iCanQuit or QuitGuide in the past, or having a housemate already enrolled were excluded. All participants provided informed consent. The Fred Hutchinson Cancer Center Institutional Review Board approved all study procedures (Ethic Approval Code: FHIRB0008317; Trial Registration: ClinicalTrials.gov identifier: NCT02724462).

Adults who were both interested and eligible to participate in the trial were randomly assigned one-to-one to receive either iCanQuit or QuitGuide for a duration of 12 months. Randomization was stratified by smoking intensity (≤20 vs. ≥21 cigarettes per day), minority race or ethnic background (non-Hispanic White race or Hispanic ethnicity), education attainment (≤high school education vs. some colleges or more), and positive screening for depression (CESD-20 scale, score ≤15 vs. ≥16). Data were collected during the 3-month, 6-month, and 12-month postrandomization follow-ups between August 2017 and December 2019 through online surveys. Participants were compensated up to $105 for completing the data collection process.

### 2.2. Participants

Out of the 2,415 adults enrolled in the iCanQuit parent RCT, 64.4% (1,555/2,415) either reported having bipolar disorder or schizophrenia or screened positive for depression, generalized anxiety disorder, panic disorder, PTSD, or social anxiety disorder [48]. Recruitment occurred from May 2017 to September 2018. The primary means of recruiting participants with mental health disorders was through Facebook ads (83.3%), a survey sampling company (12.3%), referrals from friends and family (2.3%), and websites and radio (2.1%). To deter fraud, we employed CAPTCHA authentication [55], a test designed to ascertain whether the user is human or a computer program. Moreover, any duplicate or suspicious IP addresses led to ineligibility.

### 2.3. Smartphone Intervention Apps

The iCanQuit app, detailed previously [48], consists of eight levels that are based on principles of ACT. The program emphasizes two ACT core processes: accepting triggers to smoke (e.g., sensations, emotions, thoughts) and integrating life values as motivation for quitting smoking. The “Preparing to Quit” phase teaches users skills for acceptance of triggers (i.e., mindfulness and perspective taking). The app provides an “Urge Help” feature and tracking of both letting urges pass and number of cigarettes smoked. The “After You Quit” phase covers content on staying motivated and how to prevent relapse. Users are encouraged to enable push notifications on their phones. While addressing emotional triggers to smoke (e.g., feeling anxious or sad), iCanQuit's content is not specifically framed within the context of quitting smoking with a co-occurring mental health disorder.

QuitGuide is a free app based on the US Clinical Practice Guidelines (USCPG) for smoking cessation [20]. The program offers instructional materials and hands-on methods to preparing for quitting and preventing relapse. The program provides resources, techniques, and information on triggers to smoke, barriers to quitting smoking, and FDA-approved cessation medications. In contrast with iCanQuit, which focuses on using life values as motivation for quitting, QuitGuide uses education on health consequences as motivation for quitting and trigger avoidance techniques to deal with triggers to smoke. Like iCanQuit, QuitGuide's content is not specific to quitting smoking with a co-occurring mental health disorder.

### 2.4. Measures

#### 2.4.1. Baseline Characteristics

Participants reported baseline data via online surveys, including age, education, employment ethnicity, gender, income, marital status, race, sexual orientation, and zip code. Baseline smoking behavior data included: (1) daily cigarette consumption; (2) cigarette dependence level, via the Fagerström Test for Cigarette Dependence (FTCD) [56]; (3) use of other nicotine-containing tobacco products including e-cigarettes; (4) previous attempts to quit smoking, (5) self-confidence regarding achieving smoking abstinence, and (6) smoking by friends or family members. The Quick Drinking Screen was utilized to evaluate alcohol consumption [57].

#### 2.4.2. Mental Health Disorders

To screen for depression and four prevalent anxiety disorders, we included well-established and validated self-report measures. These assessments were (1) the 20-item Center for Epidemiologic Studies Depression (CES-D) scale [58], with scores ranging from 16 to 21 indicating mild to moderate depression and scores of 22 or higher indicating major depression symptoms; (2) the 7-item Generalized Anxiety Disorder (GAD-7) scale, with scores of 10 or higher indicating GAD [59]; (3) the 5-item Autonomic Nervous System Questionnaire [60], with indication of a panic disorder recorded for individuals reporting at least one panic attack in the past month in a situation where they were not in danger or the center of attention; (4) the 6-item PTSD Checklist, with scores of 14 or higher indicating PTSD [61]; and (5) the 17-item Mini-Social Phobia Inventory with scores of 6 or higher indicating social anxiety [62].

To ensure comparability to the definitions of serious mental illness used in prior research [63, 64], serious mental illness was defined using two criteria: (i) bipolar disorder or schizophrenia and (ii) bipolar disorder, schizophrenia, or major depressive disorder (MDD), with MDD defined as a CES-D score ≥22. Bipolar disorder and schizophrenia were assessed at baseline via a single NAQC-validated question [65, 66], “Do you have any of the following mental health disorders?” Responses choices were anxiety, depression, bipolar disorder, schizophrenia, alcohol abuse, or drug abuse.

#### 2.4.3. Smoking Abstinence

Self-reported smoking abstinence at 12 months was determined by the question: “When was the last time you smoked, or even tried, a cigarette?” Response choices were “earlier today,” “24 hours ago,” “2–7 days ago,” “8–30 days ago,” and “over 30 days ago” [48, 53]. A response indicating no cigarette consumption in the past 30 days was considered as achieving abstinence for the 30-day outcome. The main outcome was 30-day smoking abstinence examined with complete-case analysis and missing-as-smoking and multiple imputation sensitivity analyses. Additional cessation outcomes included (1) cessation of combustible cigarettes and e-cigarettes (e-cig); (2) cessation of all nicotine and tobacco products (i.e., combustible cigarettes, e-cigs, chewing tobacco, snus, hookahs, cigars, cigarillos, tobacco pipes, and kreteks); and (3) prolonged abstinence.

#### 2.4.4. App Engagement and Satisfaction

Google analytics was used to objectively assess app engagement. Complete treatment engagement data for up to 12 months were unavailable due to a technical error on Google Analytics' part. Consequently, we presented engagement statistics for participants with a full 6 months of available data (1,533/1,555, 98.6%). We utilized the following metrics: (1) number (no.) of sessions, (2) no. of unique days of use, and (3) average time spent per session. Participants indicated their satisfaction with their assigned app through an 11-item measure utilizing a 5-point scale (ranging from “not at all” to “very much”) at the 3-month follow-up.

#### 2.4.5. Mediation of Acceptance of Triggers to Smoke and App Engagement

Acceptance of triggers to smoke was assessed via the 27-item Avoidance and Inflexibility Scale [67], a validated tool that assesses how willing is someone to experience physical sensations, emotions, and thoughts that cue smoking [68]. Participants rate the items on a 5-point scale ranging from “Not at all” to “Very willing.” The scores are then averaged, with higher values indicating greater acceptance.

### 2.5. Statistical Analysis

In the full sample (2,415 participants), logistic regression examined the interaction between mental health disorders and app treatment for the primary cessation outcome. Among those with at least one mental health disorder (*n* = 1,555), *t*-tests and *χ*^2^-tests compared baseline sociodemographics and smoking behaviors between arms. Mental health comorbidities were examined by summing participants with one disorder also reporting or screening positive for another. Logistic regression models tested the efficacy of iCanQuit against QuitGuide for 12-month smoking cessation and app satisfaction [53]. We also used multiple imputation of missing data as sensitivity analysis, creating 10 complete datasets and pooling results using the “mice” package in R [69]. App engagement differences were assessed with linear models and negative binomial models using the “MASS” package in R [70]. We used the PROCESS macro in SAS to explore potential mediators for the interventions' impact on 12-month cessation [71], including changes in smoking-related abstinence and engagement at 6 months [72].

All analysis models were adjusted for randomization stratification factors from the parent RCT: smoking frequency, education, and belonging to minoritized racial and/or ethnic backgrounds. Positive depression screening, used to identify the analysis sample, was excluded from these adjustments. Additionally, each model considered baseline factors differing between treatment arms and associated with the outcome [73], preserving statistical power and ensuring precise 95% confidence intervals [74]. Statistical analyses were carried out using R software version 4.2.3, with two-sided tests and *α* = 0.05 set for statistical significance [75].

## 3. Results

### 3.1. Enrollment and Outcome Data Retention

**Figure 1:**
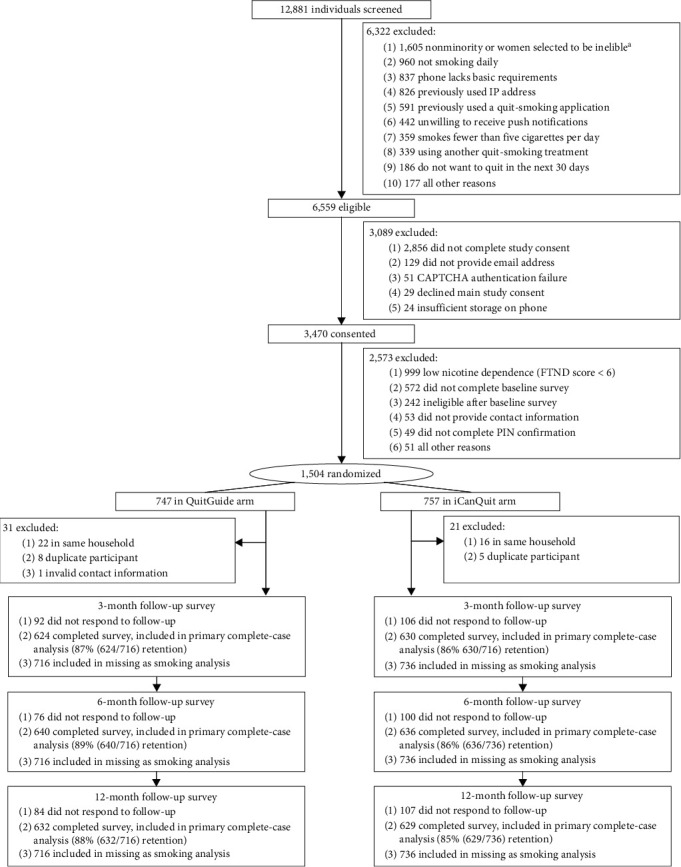
CONSORT diagram. ^a^To increase enrollment of racial/ethnic minorities and men, some nonminorities and women who were eligible for study enrollment were randomly selected to be excluded. High nicotine dependence was defined by Fagerström Test for Nicotine Dependence (FTND) scores of 6 or higher (range 0–10 = highly dependent).

The CONSORT diagram (Figure 1) visually represents the flow of the 1,555 trial participants with mental health disorders. Outcome data retention at the 12-month follow-up was 86.1%, statistically significant different by treatment arm: 83.9% for iCanQuit vs. 88.3% for QuitGuide, *P*=0.01. Given differences in retention by treatment arm, we performed missing-as-smoking and multiple imputation of the primary cessation outcome, as sensitivity analyses.

### 3.2. Participant Characteristics

Participants, recruited from all 50 US states, averaged 36.6 years of age, with 71.3% identifying as female; 19.9% identifying as lesbian, gay, bisexual, or transgender; and 71.5% identifying as single, not living with a partner, or divorced/widowed. Nearly a third (30.2%) identified as non-White and 9.3% identified as Hispanic (Table 1). Education level was 40.3% with high school diploma or lower; 52.1% were unemployed, disabled, or out of the labor force; and 41.1% had annual household incomes of $20,000 or less. Most (60.3%) had high cigarette dependence (FTCD ≥ 6), 79.9% had smoked for at least 10 years, and 26.3% were dual users of combustible and e-cigarettes. The sociodemographics and smoking behaviors at baseline were similar between participants in the iCanQuit and QuitGuide arms. Table 1 also shows that mental health disorders were evenly distributed across treatment arms.

### 3.3. Mental Health Comorbidities

In this sample of people with mental health disorders, 62.7% were classified as having a serious mental disorder (i.e., bipolar disorder, schizophrenia, or major depressive disorder). Roughly 18.8% of participants screened positive for a single mental health disorder and 18.5% screened positive for two or more mental health disorders. Over two-thirds (67.7%) of participants screened positive for PTSD, 50.2% for social anxiety, 43.2% for panic disorder, 39.7% for generalized anxiety, and 19.9% for mild to moderate depression disorder.

Mental health comorbidities were prevalent (Table 2). For instance, among those screening positive for major depression disorder (*n* = 850), 60.0% also screened positive for generalized anxiety, 47.9% for panic disorder, 85.4% for PTSD, and 65.1% for social anxiety, with 22.1% reporting bipolar disorder and 2.5% schizophrenia. Among those with bipolar disorder, 6.8% had schizophrenia, 61.4% screened positive for major depression disorder, 48.0% for generalized anxiety, 44.9% for panic disorder, 68.7% for PTSD, and 53.2% for social anxiety.

### 3.4. Smoking Cessation

Among those with any mental health disorder, iCanQuit yielded higher 12-month self-reported, 30-day cigarette smoking abstinence than QuitGuide in both complete-case analysis (24.4% vs. 20.4%; *P*=0.04) and multiple imputation sensitivity analysis (24.6% vs. 20.4%; *P*=0.04; Table 3). The missing-as-smoking sensitivity analysis was not statistically different (20.5% for iCanQuit vs. 18.1% for QuitGuide; *P*=0.16). Cessation of both combustible and e-cigarettes (20.9% vs. 16.4%; *P*=0.02), and cessation of all nicotine and tobacco products, was significantly higher in iCanQuit than QuitGuide (20.1% vs. 15.7%; *P*=0.02). No difference was observed for prolonged abstinence between arms (10.0 vs. 7.5%; *P*=0.13).

We explored cessation rate differences between apps among four groups who had: (1) bipolar disorder or schizophrenia (*n* = 318), (2) bipolar disorder, schizophrenia, or positive screening for major depression disorder (*n* = 975); (3) one positive mental health screening (292); and (4) two or more positive mental health screenings (*n* = 288). No statistically significant differences in quit rates were found between iCanQuit and QuitGuide in these subgroups (Table S1). However, a comparable effect size was observed in iCanQuit participants with bipolar disorder or schizophrenia compared with QuitGuide, albeit not statistically significant (multiple imputation: 27.1% vs. 20.9%; *P*=0.06). Although not statistically significant, for participants with one positive mental health disorder, similar differences in effect size were observed for cigarette abstinence (multiple imputation: 30.0% vs. 20.6%; *P*=0.07) and abstinence from all nicotine and tobacco products (23.8% vs. 14.7%; *P*=0.05).

### 3.5. Treatment Engagement and Satisfaction

iCanQuit users showed higher engagement than QuitGuide users with significant differences in number of logins (25.3 vs. 8.3; *P* < 0.001; Table 4), session duration (4.2 vs. 2.6 min; *P* < 0.001), and unique days of app use (16.2 vs. 6.2 days; *P* < 0.001). The mean length of app use in the first 6 months postrandomization was 51.9 days for iCanQuit vs. 55.2 days for QuitGuide and this difference was not statistically significant (*P*=0.35), suggesting that the duration of iCanQuit use was similar to that of QuitGuide. iCanQuit users also reported greater satisfaction (88.5% vs. 77.2%; *P* < 0.001) and found the app to be more useful for quitting (80.9% vs. 69.7%, *P* < 0.001). They were also more likely to recommend iCanQuit for quitting (82.0% vs. 70.6%; *P* < 0.001) and felt the app was made for them (80.4% vs. 65.0%; *P* < 0.001).

### 3.6. Mediators of Intervention Efficacy on Cessation

As hypothesized, the iCanQuit app functioned by enhancing acceptance of internal cues for smoking (Table 5) when compared to the QuitGuide app (all *P* < 0.05). The results revealed an indirect impact of the intervention app on quitting cigarette smoking at 12 months that was mediated through two key hypothesized mechanisms of action: (1) acceptance of internal emotions triggering smoking (indirect effect = 0.13, 95% CI: 0.04, 0.23) and (2) app engagement, as measured by the number of logins (indirect effect = 0.07, 95% CI: 0.01, 0.19).

### 3.7. Post Hoc Analyses

We explored potential factors influencing cessation in three subgroups: (1) participants with serious mental illness (bipolar disorder, schizophrenia, or major depression disorder); (2) those with a single positive mental health screening; and (3) those with two or more positive mental health screenings [16, 83]. We compared cigarette dependence, confidence to quit smoking, app engagement, and pharmacotherapy use at 3 months among these subgroups. Cigarette dependence did not differ between mental health subgroups. While confidence in quitting showed no significant differences between subgroups, this factor correlated positively with 12-month cessation in the subgroup of individuals with bipolar disorder, schizophrenia, or major depressive disorder only (OR = 1.02; 95% CI: 1.01, 1.02).

No significant difference in app engagement was found between participants with two or more positive screenings for mental health disorders, those with serious mental illness, and those with a single positive screening (no. logins, 12.8 vs. 19.3 vs. 17.3, respectively; *P* < 0.05). App engagement was positively associated with 12-month smoking cessation in the subgroup of participants with serious mental illness (OR = 1.01; 95% CI: 1.00, 1.01, *P* < 0.001), but not in other subgroups. Pharmacotherapy usage at 3 months was reported by 24.2% of participants with serious mental illness, 23.4% of participants with one positive mental health screening, and 20.0% of participants with two or more positive mental health screenings (*P* for comparison = .27), and no significant difference in pharmacotherapy use was observed between iCanQuit and QuitGuide arms (22.1% vs. 24.4%, *P*=0.31).

## 4. Discussion

Our study is the first to demonstrate the efficacy of ACT, as delivered by the iCanQuit app, for promoting smoking cessation among adults with mental health disorders. iCanQuit outperformed QuitGuide in achieving higher 12-month quit rates for participants with any mental health disorder. Additionally, iCanQuit was more engaging and satisfying. This superiority was consistently observed across various cessation outcomes, including (1) the primary cigarette smoking cessation outcome, with both complete-case and multiple imputation analyses, (2) cessation of combustible and e-cigarettes, and (3) cessation of all nicotine and tobacco products. These results were obtained in a large community sample and thus not limited to the unique characteristics of a mental health treatment-seeking sample.

The 12-month quit rates achieved using iCanQuit in our study are remarkable, ranging from 20.9% for cessation of all nicotine and tobacco products (including e-cigarettes) to 24.4% for cigarette smoking cessation. These rates surpass prior remote interventions evaluated with people with mental health disorders and at a longer follow-up. For instance, a two-arm RCT involving a telephone-delivered smoking cessation intervention tailored for 577 mental health patients from VA hospitals [33], the 6-month quit rates were 18% for the intervention group compared to 12% for the control group (*P* < 0.01). A subsequent secondary analysis of this RCT, focused on participants with serious mental illness [34], revealed cigarette smoking abstinence rates of 17.6% for the intervention group compared to 10.6% for the control group (*P* < 0.01).

The present study, on the other hand, represents the largest known exploration of ACT-based interventions for smoking cessation among individuals with mental health disorders. Furthermore, a 24.4% quit rate for iCanQuit would yield a 20% higher impact compared to the 20.4% quit rate for QuitGuide. For every 25 adults who smoke, iCanQuit would result in one additional successful quitter compared to QuitGuide (number needed to treat = 25) [84]. Extrapolating this impact to a population level, for every 100,000 adults who smoke, an additional 4,000 individuals would quit by utilizing iCanQuit over QuitGuide.

Regarding engagement and satisfaction with the apps, iCanQuit users exhibited higher levels of engagement and reported greater satisfaction with their assigned app compared to QuitGuide users, suggesting greater acceptability of iCanQuit in this population. Additionally, iCanQuit users demonstrated greater increases in acceptance of internal cues for smoking relative to QuitGuide users. Specifically, there was an indirect impact of the treatment on cigarette smoking cessation at 12 months, mediated through two key hypothesized mechanisms of action: (1) acceptance of internal emotions triggering smoking and (2) app engagement, as measured by the number of logins. Our group has published several secondary papers affirming the consistency of these findings [49]. Specifically, iCanQuit (not QuitGuide) exhibited a mediation effect on smoking cessation through multiple indicators of engagement, leading to a change in the mean ACT-based process of acceptance of internal cues to smoke, including acceptance of cravings to smoke. The results of this paper among participants with mental health disorders further support the robustness of this mechanism of action.

Evidence suggests that lower commitment in quitting contribute to lower quit rates in individuals with mental health comorbidities [83]. However, there was no statistically significant difference observed between those with one mental health disorder and those with two or more in this study. Considering that engagement with an app-based intervention is a strong predictor of smoking cessation [49, 85, 86, 87, 88, 89], it remains plausible that reduced engagement with the iCanQuit app contributed, at least partially, to the diminished odds of quitting smoking among participants with two or more mental health disorders.

The impact of iCanQuit on smoking cessation was mediated by increased acceptance of internal emotions triggering smoking and greater app engagement. These results support our hypotheses and substantiate the findings of the iCanQuit parent RCT [49]. Importantly, these promising results were achieved without iCanQuit containing specific content on addressing the unique challenges posed by specific mental health disorders affecting smoking cessation. This implies that iCanQuit's focus on promoting acceptance of emotions triggering smoking may serve as a mechanism of action extending beyond individual symptoms associated with multiple mental disorders (e.g., acceptance of anxiety, sadness).

Our study achieved a high 86.1% retention rate at 12 months, exceeding typical rates in smoking cessation trials among individuals with mental health disorders [33, 34]. Strengths of our study include a racially/ethnically and geographically diverse sample, a prolonged follow-up period (12 months), and comprehensive data assessing various mental health disorders. Addressing a literature gap, the study included individuals traditionally excluded from clinical trials due to mental health comorbidities, offering valuable insights into psychotic and severe mental illnesses for a better understanding of smoking cessation in this vulnerable population.

Several considerations should be noted regarding our analysis. First, the reliance on secondary data has inherent limitations. Second, there was an observed differential attrition rate between arms, although mitigated with sensitivity analyses. Third, the assessment of participants' mental health disorders relied on self-reports and screening tools, and smoking cessation outcomes were self-reported, introducing participants' subjectivity and potential reporting bias. Finally, we found a similar but not statistically significant effect size when evaluating the smoking cessation outcomes according to mental health groups. It is likely that the lack of significant findings in the subgroup analyses is due to underpowered samples. Notably, the effect sizes observed in the overall results and subgroup analyses are similar, indicating consistent trends across the data, and thereby the significance of the results. For example, the odds ratio (OR) for the 30-day PPA at the 12-month follow-up, as determined by multiple imputation analyses, was 1.31 (95% CI: 1.00, 1.70) with a *P*-value of .04. Similarly, the ORs for the subgroups ranged between 1.09 and 1.65, suggesting a comparable effect size across analyses.

Improvements are needed to incorporate features that address the unique difficulties faced by individuals coping with mental health comorbidities. Tailoring smoking cessation interventions for this group, using approaches like combined behavioral and pharmacotherapy treatments or integrated treatments addressing both smoking cessation and mental health symptoms simultaneously may be crucial. Given the substantial 82.7% prevalence of mental health comorbidities in the parent study, future trials should devise effective treatments tailored for adults actively seeking smoking cessation support.

In a diverse US sample of adults with mental health disorders, smoking cessation, engagement, and satisfaction outcomes were all significantly greater with iCanQuit than QuitGuide. iCanQuit's impact on acceptance of emotions triggering smoking and higher app engagement were important mechanisms of treatment efficacy.

## Figures and Tables

**Table 1:** Baseline characteristics of participants with mental health disorders by treatment arm.

Characteristic	*N*	No. (%) or mean (SD)		
		Overall *N* = 1,555	QuitGuide *n* = 770	iCanQuit *n* = 785
Age, years	1,555	36.6 (10.4)	36.8 (10.6)	36.4 (10.2)
Female	1,555	1,108 (71.3%)	551 (71.6%)	557 (71.0%)
Race	1,555	—	—	—
American Indian or Alaska native	—	40 (2.6%)	23 (3.0%)	17 (2.2%)
Asian American	—	2 (0.1%)	1 (0.1%)	1 (0.1%)
Black or African American	—	302 (19.4%)	151 (19.6%)	151 (19.2%)
Multiracial	—	121 (7.8%)	56 (7.3%)	65 (8.3%)
Native Hawaiian or Pacific islander	—	4 (0.3%)	2 (0.3%)	2 (0.3%)
White	—	1,062 (68.3%)	525 (68.2%)	537 (68.4%)
Unknown race	—	24 (1.5%)	12 (1.6%)	12 (1.5%)
Hispanic or latino ethnicity	1,555	145 (9.3%)	72 (9.4%)	73 (9.3%)
LGBT	1,555	310 (19.9%)	148 (19.2%)	162 (20.6%)
Education	1,555	—	—	—
GED, high school diploma or less education	—	673 (43.3%)	336 (43.6%)	337 (42.9%)
Some colleges, no degree	—	584 (37.6%)	289 (37.5%)	295 (37.6%)
College degree or higher	—	298 (19.2%)	145 (18.8%)	153 (19.5%)
Employment status	1,555	—	—	—
Employed	—	810 (52.1%)	386 (50.1%)	424 (54.0%)
Unemployed	—	234 (15.0%)	109 (14.2%)	125 (15.9%)
Disabled	—	250 (16.1%)	136 (17.7%)	114 (14.5%)
Out of labor force	—	261 (16.8%)	139 (18.1%)	122 (15.5%)
Income	1,555	—	—	—
Less than $20,000/year	—	639 (41.1%)	313 (40.6%)	326 (41.5%)
$20,000–$54,999/year	—	689 (44.3%)	357 (46.4%)	332 (42.3%)
$55,000/year or more	—	227 (14.6%)	100 (13.0%)	127 (16.2%)
Married	1,555	443 (28.5%)	226 (29.4%)	217 (27.6%)
Rural residence^a, b, c, d, e, f, g, h^	1,555	359 (23.1%)	194 (25.2%)	165 (21.0%)
Serious mental illness^i^	1,555	975 (62.7%)	479 (62.2%)	496 (63.2%)
Bipolar disorder	—	309 (19.9%)	155 (20.1%)	154 (19.6%)
Schizophrenia	—	30 (1.9%)	15 (1.9%)	15 (1.9%)
Major depression disorder	—	850 (54.8%)	420 (54.5%)	430 (55.0%)
One positive mental health screening^j^	1,555	292 (18.8%)	139 (18.1%)	153 (19.5%)
2+ positive mental health screenings^j^	1,555	288 (18.5%)	152 (19.7%)	136 (17.3%)
Alcohol use				
No. of drinks per drinking day	1,501	2.0 (4.1)	1.8 (3.7)	2.2 (4.4)
Heavy drinking^k^	1,501	225 (15.0%)	99 (13.3%)	126 (16.7%)
Smoking behavior				
No. of cigarettes smoked per day	1,555	19.3 (14.7)	19.9 (16.4)	18.7 (13.0)
FTCD score^l^	1,555	5.9 (2.1)	5.9 (2.1)	5.9 (2.1)
High nicotine dependence (FTCD ≥ 6)	1,555	938 (60.3%)	458 (59.5%)	480 (61.1%)
First cigarette within 5 min of waking	1,555	842 (54.1%)	425 (55.2%)	417 (53.1%)
Smokes more than one-half pack per day	1,555	1,140 (73.3%)	572 (74.3%)	568 (72.4%)
Smokes more than 1 pack per day	1,555	322 (20.7%)	154 (20.0%)	168 (21.4%)
Smoking for 10 or more years	1,555	1,243 (79.9%)	619 (80.4%)	624 (79.5%)
Used e-cigarettes in past month	1,555	409 (26.3%)	191 (24.8%)	218 (27.8%)
Quit attempts made in the past 12 months	1,477	600 (40.6%)	309 (42.6%)	291 (38.7%)
Confidence to quit smoking^m^	1,555	62.8 (27.1)	63.3 (27.0)	62.4 (27.2)
Friend and partner smoking				
No. of close friends who smoke	1,555	2.8 (1.7)	2.7 (1.8)	2.8 (1.7)
No. of housemates who smoke	1,555	1.5 (0.9)	1.5 (1.0)	1.5 (0.8)
Living with partner who smokes	1,555	572 (36.8%)	280 (36.4%)	292 (37.2%)
Acceptance of internal cues to smoke^n^				
Sensations	1,540	3.0 (0.6)	3.0 (0.6)	3.0 (0.6)
Emotions	1,551	2.8 (0.4)	2.8 (0.4)	2.8 (0.4)
Thoughts	1,550	2.8 (0.4)	2.8 (0.4)	2.8 (0.4)
Acceptance mean score	1,538	2.9 (0.4)	2.9 (0.4)	2.9 (0.4)

*Abbreviations*. ACT, acceptance and commitment therapy; FTCD, fagerström test for cigarette dependence; LGBT, lesbian, gay, bisexual, or transgender. aTo ascertain the residential classification of participants into urban or rural areas within the US, we utilized the ‘zipcode' R library. ^b^(Breen [76]) to link participants' Zip codes to geographic locations. The classification was based on Rural-Urban Commuting AREA (RUCA) codes at the sub-county level. ^c^(U.S. Department of Agriculture [77]). Guided by literature, RUCA codes ranging from 1 to 3 were designated as urban, whereas codes 4 to 10 were classified as rural. ^d, e, f, g, h^(Brooks et al. [78]; Chen et al. [79]; Larson et al. [80]; Ratcliffe et al. [81]; Unger et al. [82]). ^i^Presence of bipolar disorder or schizophrenia was self-reported via question: “Do you have any of the following mental health disorders?” With check all that apply response options including anxiety disorder, depression disorder, bipolar disorder, schizophrenia, alcohol abuse disorder, drug abuse disorder, or none of the above. Participants who screened positive for experiencing major depression symptoms via CESD-20 threshold ≥22 were also regarded as having a serious mental illness. ^j^Positive screening results for depression via CESD-20; score ranging from 16 to 21. Positive screening results for generalized anxiety via GAD-7; threshold ≥10. Positive screening results for panic disorder via ANSQ. A positive screening was recorded for individuals reporting at least one panic attack in the past month, provided that at least one of these incidents occurred in a situation in which they were not in danger or the center of attention. Positive screening results for PTSD via PCL-6 checklist; threshold ≥14. Positive screening results for social anxiety via mini-SPIN; threshold ≥6. ^k^Heavy drinking is defined as four or more drinks on a typical drinking day for women and five or more drinks on a typical drinking day for men within the past 30 days. ^l^Fagerström Test for Cigarette Dependence score. ^m^Range, 0–100, where 0 indicates not at all confident and 100 indicates extremely confident. ^n^Avoidance and Inflexibility Scale scores at baseline from 1 to 5. Higher scores indicate greater acceptance.

**Table 2:** Among participants with a mental health condition (column), the count, and frequency of having a co-occurring mental health condition (row).

	Bipolar disorder (BD) (*n* = 309)	Schizophrenia (SCZ) (*n* = 30)	Major depression (MDD) (*n* = 850)	Mild to moderate depression (MMD) (*n* = 309)	Generalized anxiety (GA) (*n* = 618)	Panic disorder (PD) (*n* = 672)	Posttraumatic stress disorder (PTSD) (*n* = 1,053)	Social anxiety (SA) (*n* = 781)
	*n*/*N* (%)							

Co-occurring mental health disorders								
BD	—	21/30 (70.0%)	188/850 (22.1%)	36/309 (11.7%)	147/618 (23.8%)	136/672 (20.2%)	211/1053 (20.0%)	164/781 (21.0%)
SCZ	21/309 (6.8%)	—	21/850 (2.5%)	3/309 (1.0%)	20/618 (3.2%)	16/672 (2.4%)	25/1053 (2.4%)	21/781 (2.7%)
MDD	188/306 (61.4%)	21/29 (72.4%)	—	—	508/614 (82.7%)	400/668 (59.9%)	723/1045 (69.2%)	552/773 (71.4%)
MMD	36/306 (11.8%)	3/29 (10.3%)	—	—	68/614 (11.1%)	103/668 (15.4%)	167/1045 (16.0%)	105/773 (13.6%)
GA	147/306 (48.0%)	20/30 (66.7%)	508/847 (60.0%)	68/307 (22.1%)	—	312/668 (46.7%)	572/1049 (54.5%)	424/773 (54.9%)
PD	136/303 (44.9%)	16/28 (57.1%)	400/835 (47.9%)	103/303 (34.0%)	312/604 (51.7%)	—	470/1033 (45.5%)	372/762 (48.8%)
PTSD	211/307 (68.7%)	25/30 (83.3%)	723/847 (85.4%)	167/308 (54.2%)	572/616 (92.9%)	470/669 (70.3%)	—	612/775 (79.0%)
SA	164/308 (53.2%)	21/30 (70.0%)	552/848 (65.1%)	105/307 (34.2%)	424/615 (68.9%)	372/669 (55.6%)	612/1049 (58.3%)	—

*Abbreviations*. %, percentage; BD, bipolar disorder; CI, confidence intervals; e-cigarettes, electronic (e)-cigarettes; GA, generalized anxiety; MDD, major depression disorder; MMD, mild to moderate depression; no., number; OR, odds ratio; PD, panic disorder; PPA, point prevalence abstinence, PTSD, posttraumatic stress disorder; SA, social anxiety; and SCZ, schizophrenia.

**Table 3:** Cessation outcomes at 12 months by treatment arm for participants with any mental health condition^a^.

	No. (%)			OR (95% CI)	*P*-value^b^
	Overall *N* = 1,555	QuitGuide *n* = 770	iCanQuit *n* = 785		
Primary cessation outcome variables					
30-day PPA from cigarettes, complete case^c^	300/1,339 (22.4%)	139/680 (20.4%)	161/659 (24.4%)	1.31 (1.00, 1.70)	0.04
30-day PPA from cigarettes, multiple imputation^c^	3,503/15,550 (22.5%)	1574/7700 (20.4%)	1,929/7,850 (24.6%)	1.30 (1.01, 1.67)	0.04
30-day PPA from cigarettes, missing-as-smoking^c^	300/1,555 (19.3%)	139/770 (18.1%)	161/785 (20.5%)	1.20 (0.93, 1.55)	0.16
Secondary cessation outcome variables					
30-day PPA from cigarettes, missing-as-smoking^c^	300/1,555 (19.3%)	139/770 (18.1%)	161/785 (20.5%)	1.20 (0.93, 1.55)	0.16
30-day PPA from combustible and e-cigarettes^c^	250/1,340 (18.7%)	112/681 (16.4%)	138/659 (20.9%)	1.39 (1.05, 1.85)	0.02
30-day PPA from all nicotine and tobacco products^c,d^	239/1,339 (17.8%)	107/681 (15.7%)	132/658 (20.1%)	1.40 (1.05, 1.87)	0.02
Prolonged abstinence from cigarettes^e^	95/1,090 (8.7%)	42/560 (7.5%)	53/530 (10.0%)	1.38 (0.90, 2.11)	0.13

*Abbreviations*. %, percentage; CI, confidence intervals; e-cigarettes, electronic (e)-cigarettes; no., number; OR, odds ratio; and PPA, point prevalence abstinence. ^a^Any mental health condition includes reporting bipolar disorder or schizophrenia, or screening positive for major depression disorder, mild to moderate depression disorder, generalized anxiety, panic disorder, PTSD, or social anxiety disorder. ^b^All models were adjusted for factors used in stratified randomization, except for positive screening for depression. These factors included smoking frequency, education, and minority race/ethnicity backgrounds. ^c^ Additional covariate is number of alcoholic drinks on a typical drinking day. ^d^Including any kind of e-cigarettes or vaping, chewing tobacco, snus, hookahs, cigars, cigarillos, tobacco pipes, and kreteks. ^e^Prolonged abstinence is defined as no smoking since 3-months postrandomization, using self-reported date of last cigarette.

**Table 4:** Treatment engagement and satisfaction with the assigned smartphone application among participants with any mental health condition.

Variable	*N*	Overall *N* = 1,555	QuitGuide *n* = 770	iCanQuit *n* = 785	Incidence rate ratio, point estimate, or odds ratio (95% CI)	*P*-value^a^
Engagement at 6 months						
Number of logins, mean (SD)^b^	1,533	16.9 (43.3)	8.3 (28.4)	25.3 (52.8)	IRR: 3.12 (2.72, 3.59)	<0.001
Median (IQR)	—	5 (2–15)	4 (1–9)	7 (2–25)	—	—
Time per session, minutes, mean (SD)	1,382	3.4 (4.3)	2.6 (2.7)	4.2 (5.4)	PE: 1.5 (1.1, 2.0)	<0.001
Median (IQR)	—	2.2 (1.3–3.7)	1.9 (1.1–3.1)	2.6 (1.4–4.5)	—	—
Number of unique days of use	1,533	11.2 (21.4)	6.2 (9.1)	16.2 (27.9)	IRR: 2.61 (2.30, 2.96)	<0.001
Median (IQR)	—	4 (2–11)	4 (1–8)	5 (2–16)	—	—
Satisfaction at 3 months, No. (%)						
Satisfied with assigned app	1,250	1,034 (82.7%)	495 (77.2%)	539 (88.5%)	OR: 2.28 (1.67, 3.12)	<0.001
App was useful for quitting	1,251	940 (75.1%)	446 (69.7%)	494 (80.9%)	OR: 1.85 (1.42, 2.41)	<0.001
Would recommend assigned app	1,281	976 (76.2%)	460 (70.6%)	516 (82.0%)	OR: 1.93 (1.48, 2.53)	<0.001
Felt app was made for me	1,229	892 (72.6%)	404 (65.0%)	488 (80.4%)	OR: 2.24 (1.72, 2.90)	<0.001

*Abbreviations*. %, percentage; CI, confidence intervals; IRR, incidence rate ratio; IQR, interquartile range, PE, point estimate; OR, odds ratio; and SD, standard deviation. ^a^All models were adjusted for factors used in stratified randomization, except for positive screening for depression. These factors included daily smoking frequency, education, and minority race/ethnicity backgrounds. ^b^Additional covariate is heavy drinking.

**Table 5:** Mediation of the treatment effect on 12-month abstinence from combustible cigarettes by baseline to 3-month changes in ACT-based acceptance processes and by engagement with the apps.

Mediator	*N*	Mean (SD)			Point estimate or incidence rate ratio for difference (95% CI)	*P*-value	Estimate of indirect effect (95% CI)
		Overall *N* = 1,555	QuitGuide *n* = 770	iCanQuit *n* = 785			
Acceptance of internal cues to smoke							
Physical sensations^a^	1,262	0.16 (0.74)	0.12 (0.68)	0.20 (0.78)	PE: 0.08 (0.01, 0.15)	0.02	0.02 (−0.01, 0.06)
Emotions	1,281	0.14 (0.64)	0.07 (0.57)	0.21 (0.70)	PE: 0.13 (0.07, 0.19)	<0.001	0.13 (0.04, 0.23)*⁣*^*∗*^
Thoughts	1,281	0.13 (0.61)	0.06 (0.55)	0.20 (0.66)	PE: 0.13 (0.07, 0.18)	<0.001	0.01 (−0.05, 0.08)
App engagement^b^							
Number of logins in first 6 months^c^	1,533	16.9 (43.3)^d^	8.3 (28.4)^e^	25.3 (52.8)^f^	IRR: 3.12 (2.72, 3.59)	<0.001	0.07 (0.01, 0.19)*⁣*^*∗*^

*Abbreviations*. %, percentage; CI, confidence intervals; ACT, acceptance and commitment therapy; IRR, incidence rate ratio; PE, point estimate; SD, standard deviation. ^a^All changes in acceptance scores calculated as value at 3-months minus baseline value. ^b^A full 6-months of utilization data from Google Analytics were available for *n* = 1533/1555, 98.6%. ^c^Additional covariate is heavy drinking. ^d^Median (IQR): 5 (2–15). ^e^Median (IQR): 4 (1–9). ^f^Median (IQR): 7 (2–25). *⁣*^*∗*^*P* < 0.05.

## Data Availability

The data will be shared upon reasonable request to Jonathan B. Bricker at jbricker@fredhutch.org. iCanQuit is freely accessible to the public and no licensing fees apply.

## Abbreviations

%: Percentage
ACT: Acceptance and commitment therapy
BD: Bipolar disorder
CI: 95% confidence interval
e-cigarettes: Electronic (e)-cigarettes
FTCD: Fagerström Test for Cigarette Dependence
GA: Generalized anxiety
GED: General education development
LGBT: Lesbian, gay, bisexual, or transgender
MDD: Major depression disorder
MMDD: Mild to moderate depression disorder
no.: Number
OR: Odds ratio
PD: Panic disorder
PPA: Point-prevalence abstinence
PTSD: Posttraumatic stress disorder
RCT: Randomized controlled trial
SA: Social anxiety
SCZ: Schizophrenia
US: United States
USCPG: US Clinical Practice Guidelines.

## Supplementary Materials

Supplementary Materials Table S1: smoking cessation outcomes at 12 months by treatment arm in four mental health groups of participants.
