# Supplementary material for: Can an Acceptance and Commitment Therapy-Based Smartphone App Help Individuals with Mental Health Disorders Quit Smoking?
Source: Depress Anxiety. Author manuscript; Available in PMC 2025 Jan 22. (PMC11753409; doi:10.1155/2024/1055801)
Supplement: Supplementary file 1 [file NIHMS2044581-supplement-Supplementary_Material.docx]

| **Supplementary Table 1.** Smoking cessation outcomes at 12 months by treatment arm in four mental health groups of participants. |  |  |  |  |  |  |
| --- | --- | --- | --- | --- | --- | --- |
|  |  | No. (%) |  |  |  |  |
|  | N | Overall N = 1555 | QuitGuide n = 770 | iCanQuit n = 785 | OR (95% CI) | P value^ a^ |
| **30-d PPA from cigarettes, multiple imputation** |  |  |  |  |  |  |
| Bipolar disorder or schizophrenia | 3180 | 761/3180 (23.9%) | 339/1620 (20.9%) | 422/1560 (27.1%) | 1.27 (0.99, 1.64) | .06 |
| Bipolar disorder, schizophrenia, or major depression disorder | 9750 | 2059/9750 (21.1%) | 930/4790 (19.4%) | 1129/4960 (22.8%) | 1.22 (0.87, 1.71) | .24 |
| 1 Positive Mental Health Screening ^b^ | 2920 | 746/2920 (25.5%) | 287/1390 (20.6%) | 459/1530 (30.0%) | 1.65 (0.95, 2.86) | .07 |
| 2+ Positive Mental Health Screenings ^b^ | 2880 | 698/2880 (24.2%) | 357/1520 (23.5%) | 341/1360 (25.1%) | 1.09 (0.62, 1.92) | .76 |
| **30-d PPA from cigarettes, complete case** |  |  |  |  |  |  |
| Bipolar disorder or schizophrenia | 318 | 66/276 (23.9%) | 29/142 (20.4%) | 37/134 (27.6%) | 1.50 (0.85, 2.64) | .16 |
| Bipolar disorder, schizophrenia, or major depression disorder | 975 | 174/829 (21.0%) | 80/416 (19.2%) | 94/413 (22.8%) | 1.25 (0.89, 1.75) | .19 |
| 1 Positive Mental Health Screening ^b^ | 292 | 65/256 (25.4%) | 27/129 (20.9%) | 38/127 (29.9%) | 1.61 (0.91, 2.85) | .10 |
| 2+ Positive Mental Health Screenings ^b^ | 288 | 61/254 (24.0%) | 32/135 (23.7%) | 29/119 (24.4%) | 1.04 (0.58, 1.87) | .88 |
| **30-d PPA from cigarettes, missing-as-smoking** |  |  |  |  |  |  |
| Bipolar disorder or schizophrenia | 318 | 66/318 (20.8%) | 29/162 (17.9%) | 37/156 (23.7%) | 1.42 (0.81, 2.46) | .21 |
| Bipolar disorder, schizophrenia, or major depression disorder | 975 | 174/975 (17.8%) | 80/479 (16.7%) | 94/496 (19.0%) | 1.16 (0.84, 1.62) | .36 |
| 1 Positive Mental Health Screening ^b^ | 292 | 65/292 (22.3%) | 27/139 (19.4%) | 38/153 (24.8%) | 1.36 (0.78, 2.39) | .27 |
| 2+ Positive Mental Health Screenings ^b, c^ | 288 | 61/288 (21.2%) | 32/152 (21.1%) | 29/136 (21.3%) | 1.09 (0.61, 1.94) | .77 |
| **30-d PPA from all nicotine and tobacco products** ^**d**^ |  |  |  |  |  |  |
| Bipolar disorder or schizophrenia | 318 | 55/275 (20.0%) | 24/142 (16.9%) | 31/133 (23.3%) | 1.51 (0.82, 2.77) | .18 |
| Bipolar disorder, schizophrenia, or major depression disorder | 975 | 140/829 (16.9%) | 62/416 (14.9%) | 78/413 (18.9%) | 1.34 (0.93, 1.94) | .11 |
| 1 Positive Mental Health Screening ^b^ | 292 | 49/255 (19.2%) | 19/129 (14.7%) | 30/126 (23.8%) | 1.86 (0.98, 3.53) | .05 |
| 2+ Positive Mental Health Screenings ^b^ | 288 | 50/255 (19.6%) | 26/136 (19.1%) | 24/119 (20.2%) | 1.05 (0.56, 1.95) | .88 |
| **30-d PPA from both combustible and e-cigarettes** |  |  |  |  |  |  |
| Bipolar disorder or schizophrenia | 318 | 59/276 (21.4%) | 25/142 (17.6%) | 34/134 (25.4%) | 1.61 (0.89, 2.91) | .11 |
| Bipolar disorder, schizophrenia, or major depression disorder | 975 | 147/830 (17.7%) | 65/416 (15.6%) | 82/414 (19.8%) | 1.35 (0.94, 1.93) | .10 |
| 1 Positive Mental Health Screening ^b^ | 292 | 51/255 (20.0%) | 21/129 (16.3%) | 30/126 (23.8%) | 1.65 (0.88, 3.08) | .11 |
| 2+ Positive Mental Health Screenings ^b^ | 288 | 52/255 (20.4%) | 26/136 (19.1%) | 26/119 (21.8%) | 1.17 (0.63, 2.15) | .62 |
| **Prolonged abstinence from cigarettes** ^**e**^ |  |  |  |  |  |  |
| Bipolar disorder or schizophrenia | 318 | 18/212 (9.5%) | 7/107 (6.5%) | 11/105 (10.5%) | 1.62 (0.60, 4.39) | .34 |
| Bipolar disorder, schizophrenia, or major depression disorder | 975 | 54/658 (8.2%) | 24/331 (7.3%) | 30/327 (9.2%) | 1.31 (0.75, 2.30) | .34 |
| 1 Positive Mental Health Screening ^b^ | 292 | 18/218 (8.3%) | 6/112 (5.4%) | 12/106 (11.3%) | 2.12 (0.76, 5.93) | .15 |
| 2+ Positive Mental Health Screenings ^b^ | 288 | 23/214 (10.7%) | 12/117 (10.3%) | 11/97 (11.3%) | 1.11 (0.47, 2.65) | .81 |

Abbreviations: %, percentage; CI, confidence intervals; e-cigarettes, electronic (e)-cigarettes; no., number; OR, odds ratio; PPA, point prevalence abstinence.

^a ^All models were adjusted for factors used in stratified randomization, except for positive screening for depression symptoms. These factors included daily smoking frequency, education, and minority race/ethnicity backgrounds.

^b ^Does not include participants who reported bipolar disorder, schizophrenia, or who had positive screening for major depression disorder symptoms.

^c ^Additional covariate is Fagerström Test for Cigarette Dependence (FTCD) score.

^d ^Including any kind of e-cigarettes or vaping, chewing tobacco, snus, hookahs, cigars, cigarillos, tobacco pipes, and kreteks.

^e ^Prolonged abstinence is defined as no smoking since 3-months post-randomization, using self-reported date of last cigarette.
